# Di-μ-aqua-bis­[aqua­(2,2′-bi­pyridine)(4-nitro­benzoato)cobalt(II)] bis­(4-nitro­benzoate)

**DOI:** 10.1107/S2414314620007968

**Published:** 2020-06-19

**Authors:** Bikshandarkoil R. Srinivasan, Sarvesh S. Harmalkar, Luann R. D’Souza, Sunder N. Dhuri

**Affiliations:** a School of Chemical Sciences, Goa University, Goa 403206, India; University of Aberdeen, Scotland

**Keywords:** crystal structure, bimetallic complex, divalent-metal 4-nitro­benzoate, hydrogen bonds

## Abstract

The title compound, [Co_2_(C_7_H_4_NO_4_)_2_(C_10_H_8_N_2_)_2_(H_2_O)_4_](C_7_H_4_NO_4_)_2_, consists of a centrosymmetric bimetallic complex charge-balanced by free 4-nitro­benzoate anions. The Co^II^ ion exhibits a distorted *cis*-CoN_2_O_4_ octa­hedral coordination environment and the Co⋯Co separation is 3.326 (2) Å. In the crystal, the dications and anions are linked by O—H⋯O and C—H⋯O hydrogen bonds.

## Abstract 2

The title compound consists of a centrosymmetric bimetallic complex charge-balanced by free 4-nitro­benzoate anions. Each Co^II^ ion exhibits a distorted *cis*-CoN_2_O_4_ octa­hedral coordination environment. In the crystal, the dications and anions are linked by O—H⋯O and C—H⋯O hydrogen bonds.

## Graphical abstract

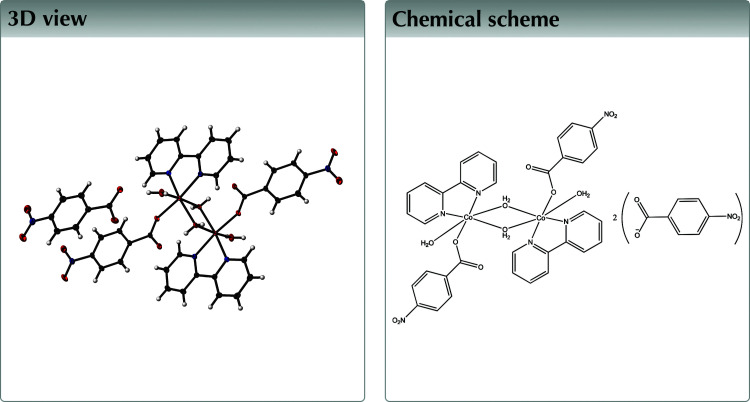

## Structure description

As part of an ongoing research program we are investigating the structural aspects of mixed-ligand compounds of divalent-metal 4-nitro­benzoates. Recently we described the structure of [Co(H_2_O)_2_(DMSO)_2_(C_7_H_4_NO_4_)](C_7_H_4_NO_4_) **2** (DMSO = di­methyl­sulfoxide; C_7_H_4_NO_4_ = 4-nitro­benzoate) containing a bidentate as well as a free 4-nitro­benzoate anion (Srinivasan *et al.*, 2020 ▸). Our attempts to replace the *cis*-aqua ligands of **2** with 2,2′-bi­pyridine has resulted in the isolation of the di­aqua-bridged title dinuclear compound. The Cambridge Structural Database (CSD, version 5.40, update September 2019; Groom *et al.*, 2016 ▸) lists the structures of several cobalt 4-nitro­benzoates: of these, more than a dozen are mononuclear cobalt compounds (Srinivasan *et al.*, 2004 ▸, 2020 ▸; Chakravorty *et al.*, 2011 ▸) while only four dinuclear compounds of 4-nitro­benzoate are known to date (Singh *et al.*, 2007 ▸; Jung *et al.*, 2009 ▸; Yang *et al.*, 2011 ▸; Wang & Qi, 2014 ▸). The title compound is a new addition to the list of dimeric cobalt 4-nitro­benzoates.

**Figure 1:**
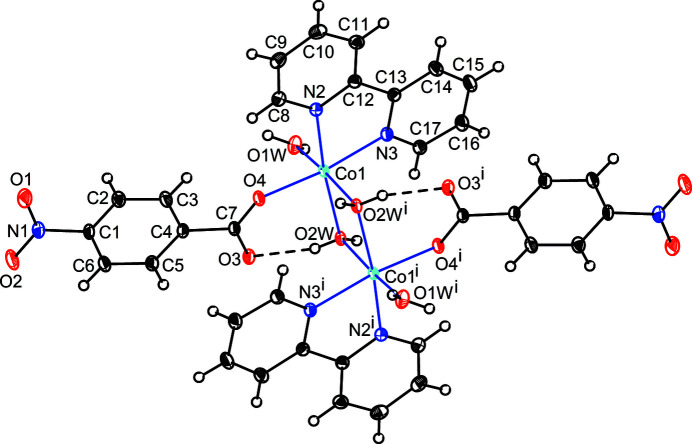
The dinuclear dication in **1** with displacement ellipsoids drawn at the 50% probability level. Intramolecular hydrogen bonds are shown as broken lines [Symmetry code: (i) 1 – *x*, 1 – *y*, 1 – *z*.]

The structure of the title compound, **1**, consists of a crystallographically unique cobaltous ion and a 2,2′-bi­pyridine mol­ecule, two crystallographically independent 4-nitro­benzoate ions and two unique aqua ligands (one terminal, one bridging). The Co^II^ ion, one 4-nitro­benzoate ion, one 2,2′-bi­pyridine mol­ecule and each of a terminal and bridging water mol­ecule build up one half of a dimeric dicationic species [Co_2_(H_2_O)_2_(C_10_H_8_N_2_)_2_(C_7_H_4_NO_4_)_2_(μ_2_-H_2_O)_2_]^2+^, the other half being generated by inversion symmetry (Fig. 1 ▸). The crystallographic inversion centre is situated at the midpoint of the line connecting the Co^II^ atoms in the dimer. A charge-balancing 4-nitro­benzoate ion completes the structure.

In the centrosymmetric dimer, each Co^II^ ion exhibits a distorted octa­hedral environment and is bonded to a terminal aqua ligand, a monodentate 4-nitro­benzoate ligand disposed *cis* to the terminal aqua ligand and a bidentate 2,2′-bi­pyridine mol­ecule. A pair of *cis*-aqua ligands bridges the metal centres and completes the hexa-coordination around the metal ions resulting in a Co⋯Co(1 – *x*, 1 – *y*, 1 – *z*) separation of 3.326 (2) Å. It is inter­esting to note that in three of the four known dinuclear cobalt compounds (Singh *et al.*, 2007 ▸; Yang *et al.*, 2011 ▸; Wang & Qi, 2014 ▸), the 4-nitro­benzoate anion functions as a monodentate ligand as in the title compound. One example each of a dinuclear (Jung *et al.*, 2009 ▸) and a tetra­nuclear cobalt compound (Dimitrou *et al.*, 2001 ▸) is known where the 4-nitro­benzoate ion functions as a symmetric bridging ligand.

The geometric parameters of **1** are in their normal ranges and are in agreement with reported data (Srinivasan *et al.*, 2020 ▸). The Co—O_w_ (w = water) bonds [2.0743 (10) and 2.1617 (9) Å] are elongated as compared to the Co—O_c_ (c = carboxyl­ate) distance, which is the shortest at 2.0494 (9) Å. The *cis*-O—Co—O and N—Co—N bond angles range between 77.97 (4) and 100.02 (4)°, while the *trans* bond angles deviate from ideal values, indicating a distortion of the {CoN_2_O_4_} octa­hedron.

**Figure 2:**
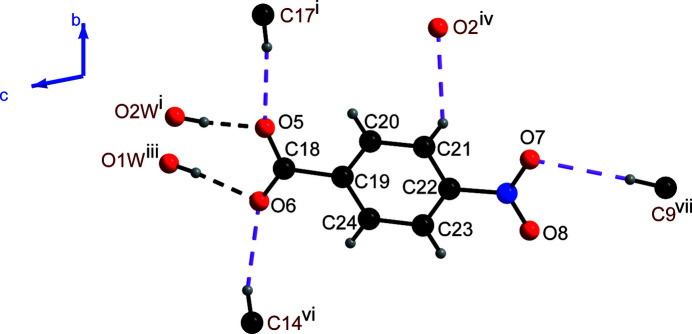
The hydrogen-bonding scheme around the 4-nitro­benzoate anion showing the O—H⋯O and C—H⋯O hydrogen bonds as dashed lines. For symmetry codes see Table 1 ▸.

**Figure 4:**
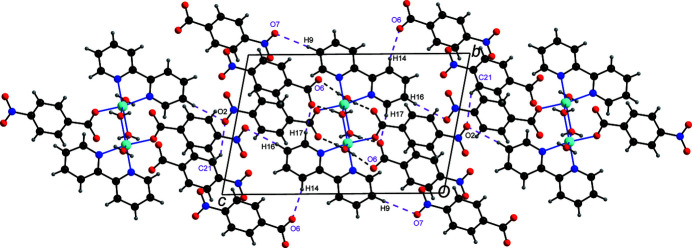
The hydrogen-bonding scheme around the dication showing its linking with eight anions and two cations *via* O—H⋯O and C—H⋯O hydrogen bonds.

All of the H atoms attached to the aqua ligands, and five of the other H atoms *viz*. H9, H14, H16, H17 and H21 bonded to C9, C14, C16, C17 and C21, respectively, function as hydrogen-bond donors, while the oxygen atoms O2, O3, O5, O6 and O7 of the 4-nitro­benzoate ions function as acceptors, resulting in a total of four O—H⋯O and five C—H⋯O hydrogen bonds (Table 1 ▸). Each free 4-nitro­benzoate anion is linked with four symmetry-related dications with the aid of two O—H⋯O hydrogen bonds and four C—H⋯O hydrogen bonds (Figs. 2 ▸ and 3 ▸). Each of the dinuclear dicobalt dicationic species is linked with two symmetry-related dications and eight symmetry-generated anions (Fig. 4 ▸), resulting in a three-dimensional supra­molecular network.

## Synthesis and crystallization

Crystals of **2** (0.0292 g, 0.05 mmol) were taken in DMSO (3 ml) to obtain a purple solution. 2,2′-Bi­pyridine (0.0078 g, 0.05 mmol) was dissolved in DMSO (3 ml) in a separate beaker and then was added dropwise to the cobalt solution with continuous swirling. The pale-orange solution thus obtained was left undisturbed at room temperature. After to days, dark-orange blocks of **1** started forming in the solution, which were isolated by filtration and air dried. Yield 60%.

## Refinement

Crystal data, data collection and structure refinement details are summarized in Table 2 ▸.

## Supplementary Material

Crystal structure: contains datablock(s) I, global. DOI: 10.1107/S2414314620007968/hb4348sup1.cif

Structure factors: contains datablock(s) I. DOI: 10.1107/S2414314620007968/hb4348Isup2.hkl

CCDC reference: 2009578

Additional supporting information:  crystallographic information; 3D view; checkCIF report

## Figures and Tables

**Figure 3:**
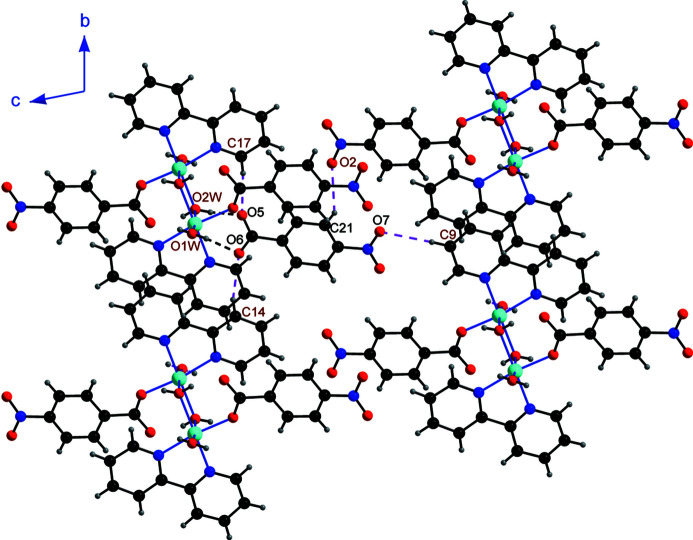
Environment of the anion, showing its hydrogen bonds to four symmetrically related dications *via* O—H⋯O and C—H⋯O bonds.

**Table 1:** Hydrogen-bond geometry (Å, °)

*D*—H⋯*A*	*D*—H	H⋯*A*	*D*⋯*A*	*D*—H⋯*A*
O2*W*—H2*B*⋯O5^i^	0.84 (2)	1.67 (2)	2.5101 (13)	173 (2)
O2*W*—H2*A*⋯O3	0.79 (2)	1.88 (2)	2.6483 (13)	164 (2)
O1*W*—H1*B*⋯O3^ii^	0.82 (2)	2.04 (2)	2.8477 (14)	174 (2)
O1*W*—H1*A*⋯O6^iii^	0.81 (2)	1.88 (2)	2.6803 (14)	171 (2)
C21—H21⋯O2^iv^	0.93	2.48	3.2219 (17)	137
C17—H17⋯O5^i^	0.93	2.24	3.1679 (16)	172
C16—H16⋯O2^v^	0.93	2.57	3.4644 (17)	160
C14—H14⋯O6^vi^	0.93	2.41	3.3126 (16)	164
C9—H9⋯O7^vii^	0.93	2.64	3.5467 (18)	164

Symmetry codes: (i) 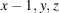 ; (ii) 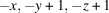 ; (iii) 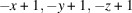 ; (iv) 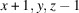 ; (v) 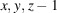 ; (vi) 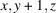 ; (vii) 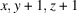 .

**Table 2:** Experimental details

Crystal data	
Chemical formula	[Co_2_(C_7_H_4_NO_4_)_2_(C_10_H_8_N_2_)_2_(H_2_O)_4_](C_7_H_4_NO_4_)_2_
*M*_r_	1166.74
Crystal system, space group	Triclinic, *P* 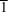
Temperature (K)	296
*a*, *b*, *c* (Å)	7.2747 (5), 10.4927 (8), 16.3560 (12)
α, β, γ (°)	97.735 (2), 102.840 (2), 102.607 (2)
*V* (Å^3^)	1165.70 (15)
*Z*	1
Radiation type	Mo *K*α
μ (mm^−1^)	0.81
Crystal size (mm)	0.45 × 0.32 × 0.21

Data collection	
Diffractometer	Bruker D8 Quest eco
Absorption correction	Multi-scan (*SADABS*; Krause *et al.*, 2015 ▸)
No. of measured, independent and observed [*I* > 2σ(*I*)] reflections	26747, 5763, 5312
*R*_int_	0.025
(sin θ/λ)_max_ (Å^−1^)	0.666

Refinement	
*R*[*F* ^2^ > 2σ(*F* ^2^)], *wR*(*F* ^2^), *S*	0.025, 0.064, 1.05
No. of reflections	5763
No. of parameters	368
H-atom treatment	H atoms treated by a mixture of independent and constrained refinement
Δρ_max_, Δρ_min_ (e Å^−3^)	0.36, −0.31

Computer programs: *APEX3* and *SAINT* (Bruker, 2019 ▸), *SHELXT2014/5* (Sheldrick, 20015*a* ▸), *SHELXL2018* (Sheldrick, 2015*b* ▸), *DIAMOND* (Brandenburg, 1999 ▸) and *shelXle* (Hübschle *et al.*, 2011 ▸).

## full crystallographic data

### Crystal data

**Table:**

[Co_2_(C_7_H_4_NO_4_)_2_(C_10_H_8_N_2_)_2_(H_2_O)_4_](C_7_H_4_NO_4_)_2_	*Z* = 1
*M**_r_* = 1166.74	*F*(000) = 598
Triclinic, *P*1	*D*_x_ = 1.662 Mg m^−^^3^
*a* = 7.2747 (5) Å	Mo *K*α radiation, λ = 0.71073 Å
*b* = 10.4927 (8) Å	Cell parameters from 9978 reflections
*c* = 16.3560 (12) Å	θ = 2.9–28.2°
α = 97.735 (2)°	µ = 0.81 mm^−^^1^
β = 102.840 (2)°	*T* = 296 K
γ = 102.607 (2)°	Block, orange
*V* = 1165.70 (15) Å^3^	0.45 × 0.32 × 0.21 mm

### Data collection

**Table:**

Bruker D8 Quest eco diffractometer	5312 reflections with *I* > 2σ(*I*)
Radiation source: Sealed Tube	*R*_int_ = 0.025
φ and ω scans	θ_max_ = 28.3°, θ_min_ = 2.6°
Absorption correction: multi-scan (SADABS; Krause *et al.*, 2015)	*h* = −9→9
	*k* = −13→13
26747 measured reflections	*l* = −21→21
5763 independent reflections	

### Refinement

**Table:**

Refinement on *F*^2^	0 restraints
Least-squares matrix: full	Hydrogen site location: mixed
*R*[*F*^2^ > 2σ(*F*^2^)] = 0.025	H atoms treated by a mixture of independent and constrained refinement
*wR*(*F*^2^) = 0.064	*w* = 1/[σ^2^(*F*_o_^2^) + (0.0255*P*)^2^ + 0.6838*P*] where *P* = (*F*_o_^2^ + 2*F*_c_^2^)/3
*S* = 1.05	(Δ/σ)_max_ = 0.003
5763 reflections	Δρ_max_ = 0.36 e Å^−^^3^
368 parameters	Δρ_min_ = −0.31 e Å^−^^3^

### Special details

**Table:**

Geometry. All esds (except the esd in the dihedral angle between two l.s. planes) are estimated using the full covariance matrix. The cell esds are taken into account individually in the estimation of esds in distances, angles and torsion angles; correlations between esds in cell parameters are only used when they are defined by crystal symmetry. An approximate (isotropic) treatment of cell esds is used for estimating esds involving l.s. planes.

### Fractional atomic coordinates and isotropic or equivalent isotropic displacement parameters (Å^2^)

**Table:**

	*x*	*y*	*z*	*U*_iso_*/*U*_eq_	
Co1	0.43361 (2)	0.63922 (2)	0.52457 (2)	0.01063 (5)	
O1	0.43137 (17)	0.71403 (11)	1.06326 (6)	0.0261 (2)	
N1	0.32901 (17)	0.60682 (12)	1.01984 (7)	0.0180 (2)	
C1	0.32850 (19)	0.57702 (13)	0.92928 (8)	0.0147 (2)	
O2	0.22558 (18)	0.52407 (11)	1.04780 (7)	0.0289 (2)	
N2	0.61203 (16)	0.83416 (10)	0.57038 (7)	0.0131 (2)	
C2	0.45366 (19)	0.66787 (13)	0.89966 (8)	0.0168 (3)	
H2	0.539777	0.742960	0.936466	0.020*	
O3	0.19021 (13)	0.40968 (9)	0.61539 (6)	0.01502 (18)	
N3	0.44762 (16)	0.70799 (11)	0.41050 (7)	0.0130 (2)	
C3	0.44682 (19)	0.64358 (13)	0.81350 (8)	0.0161 (2)	
H3	0.529735	0.703100	0.792083	0.019*	
O4	0.43133 (13)	0.59742 (9)	0.64323 (6)	0.01478 (18)	
N4	0.82411 (18)	0.08005 (12)	−0.06413 (8)	0.0207 (2)	
C4	0.31743 (18)	0.53122 (12)	0.75861 (8)	0.0129 (2)	
C5	0.1950 (2)	0.44082 (13)	0.79086 (8)	0.0169 (3)	
H5	0.109775	0.365050	0.754405	0.020*	
O5	1.07575 (15)	0.36007 (10)	0.32867 (6)	0.0211 (2)	
C6	0.1994 (2)	0.46313 (13)	0.87707 (8)	0.0180 (3)	
H6	0.118041	0.403405	0.899036	0.022*	
O6	0.85644 (15)	0.18061 (9)	0.33776 (6)	0.0203 (2)	
C7	0.31025 (18)	0.51044 (12)	0.66454 (8)	0.0127 (2)	
O7	0.91826 (18)	0.15271 (12)	−0.10126 (7)	0.0317 (3)	
C8	0.6889 (2)	0.89288 (13)	0.65249 (8)	0.0167 (3)	
H8	0.650756	0.848993	0.694104	0.020*	
O8	0.70001 (17)	−0.02340 (11)	−0.10076 (7)	0.0297 (2)	
C9	0.8231 (2)	1.01652 (13)	0.67843 (9)	0.0188 (3)	
H9	0.874547	1.054302	0.736112	0.023*	
C10	0.8785 (2)	1.08205 (13)	0.61629 (9)	0.0191 (3)	
H10	0.967902	1.164988	0.631700	0.023*	
C11	0.79935 (19)	1.02285 (13)	0.53093 (9)	0.0165 (2)	
H11	0.834491	1.065696	0.488378	0.020*	
C12	0.66666 (18)	0.89850 (12)	0.50973 (8)	0.0131 (2)	
C13	0.57188 (18)	0.82847 (12)	0.41994 (8)	0.0130 (2)	
C14	0.6047 (2)	0.88285 (13)	0.34951 (9)	0.0176 (3)	
H14	0.691802	0.965414	0.356946	0.021*	
C15	0.5048 (2)	0.81138 (14)	0.26838 (9)	0.0206 (3)	
H15	0.525861	0.845041	0.220521	0.025*	
C16	0.3734 (2)	0.68944 (14)	0.25880 (9)	0.0188 (3)	
H16	0.302949	0.641070	0.204804	0.023*	
C17	0.34957 (19)	0.64140 (13)	0.33163 (8)	0.0155 (2)	
H17	0.261827	0.559500	0.325489	0.019*	
C18	0.95292 (19)	0.24975 (13)	0.29757 (8)	0.0146 (2)	
C19	0.92261 (19)	0.20072 (13)	0.20274 (8)	0.0149 (2)	
C20	1.03275 (19)	0.27527 (13)	0.15766 (9)	0.0173 (3)	
H20	1.127507	0.352694	0.186407	0.021*	
O1W	0.16527 (15)	0.68171 (11)	0.51582 (7)	0.0186 (2)	
C21	1.0024 (2)	0.23507 (14)	0.07020 (9)	0.0189 (3)	
H21	1.075168	0.284846	0.039878	0.023*	
C22	0.8611 (2)	0.11911 (13)	0.02938 (9)	0.0178 (3)	
C23	0.7511 (2)	0.04133 (14)	0.07225 (9)	0.0218 (3)	
H23	0.658049	−0.036827	0.043395	0.026*	
C24	0.7839 (2)	0.08360 (14)	0.15973 (9)	0.0203 (3)	
H24	0.712092	0.032818	0.189930	0.024*	
O2W	0.30828 (13)	0.43132 (9)	0.47476 (6)	0.01185 (17)	
H1A	0.148 (3)	0.718 (2)	0.5594 (15)	0.042 (6)*	
H1B	0.066 (3)	0.650 (2)	0.4773 (14)	0.039 (6)*	
H2A	0.264 (3)	0.410 (2)	0.5123 (15)	0.045 (6)*	
H2B	0.223 (3)	0.404 (2)	0.4276 (14)	0.039 (6)*	

### Atomic displacement parameters (Å^2^)

**Table:**

	*U*^11^	*U*^22^	*U*^33^	*U*^12^	*U*^13^	*U*^23^
Co1	0.01158 (9)	0.01116 (8)	0.00889 (8)	0.00091 (6)	0.00424 (6)	0.00170 (6)
O1	0.0363 (6)	0.0240 (5)	0.0128 (5)	0.0000 (5)	0.0064 (4)	−0.0016 (4)
N1	0.0228 (6)	0.0209 (6)	0.0115 (5)	0.0064 (5)	0.0061 (4)	0.0034 (4)
C1	0.0187 (6)	0.0180 (6)	0.0092 (6)	0.0061 (5)	0.0056 (5)	0.0031 (5)
O2	0.0405 (6)	0.0285 (6)	0.0170 (5)	−0.0013 (5)	0.0154 (5)	0.0056 (4)
N2	0.0144 (5)	0.0128 (5)	0.0126 (5)	0.0034 (4)	0.0045 (4)	0.0024 (4)
C2	0.0173 (6)	0.0177 (6)	0.0128 (6)	0.0004 (5)	0.0037 (5)	0.0011 (5)
O3	0.0163 (4)	0.0161 (4)	0.0111 (4)	0.0010 (3)	0.0048 (3)	0.0008 (3)
N3	0.0136 (5)	0.0141 (5)	0.0118 (5)	0.0033 (4)	0.0047 (4)	0.0025 (4)
C3	0.0166 (6)	0.0180 (6)	0.0130 (6)	0.0003 (5)	0.0061 (5)	0.0032 (5)
O4	0.0174 (4)	0.0162 (4)	0.0112 (4)	0.0019 (4)	0.0065 (3)	0.0032 (3)
N4	0.0239 (6)	0.0204 (6)	0.0168 (6)	0.0057 (5)	0.0054 (5)	0.0002 (5)
C4	0.0139 (6)	0.0156 (6)	0.0108 (6)	0.0051 (5)	0.0049 (4)	0.0028 (5)
C5	0.0200 (6)	0.0154 (6)	0.0135 (6)	0.0003 (5)	0.0063 (5)	0.0008 (5)
O5	0.0234 (5)	0.0185 (5)	0.0136 (5)	−0.0051 (4)	−0.0005 (4)	0.0027 (4)
C6	0.0230 (7)	0.0176 (6)	0.0141 (6)	0.0014 (5)	0.0092 (5)	0.0041 (5)
O6	0.0241 (5)	0.0161 (4)	0.0205 (5)	−0.0001 (4)	0.0116 (4)	0.0012 (4)
C7	0.0139 (6)	0.0154 (6)	0.0109 (6)	0.0056 (5)	0.0053 (4)	0.0037 (5)
O7	0.0416 (7)	0.0307 (6)	0.0202 (5)	−0.0008 (5)	0.0134 (5)	0.0028 (5)
C8	0.0194 (6)	0.0161 (6)	0.0143 (6)	0.0047 (5)	0.0046 (5)	0.0016 (5)
O8	0.0345 (6)	0.0258 (5)	0.0202 (5)	−0.0017 (5)	0.0046 (5)	−0.0046 (4)
C9	0.0200 (6)	0.0174 (6)	0.0156 (6)	0.0047 (5)	0.0011 (5)	−0.0023 (5)
C10	0.0165 (6)	0.0137 (6)	0.0240 (7)	0.0017 (5)	0.0038 (5)	−0.0003 (5)
C11	0.0163 (6)	0.0135 (6)	0.0199 (6)	0.0024 (5)	0.0065 (5)	0.0036 (5)
C12	0.0126 (6)	0.0133 (6)	0.0141 (6)	0.0038 (5)	0.0042 (5)	0.0026 (5)
C13	0.0125 (6)	0.0130 (5)	0.0134 (6)	0.0027 (4)	0.0041 (5)	0.0022 (5)
C14	0.0194 (6)	0.0159 (6)	0.0173 (6)	0.0008 (5)	0.0069 (5)	0.0047 (5)
C15	0.0271 (7)	0.0216 (7)	0.0138 (6)	0.0032 (6)	0.0079 (5)	0.0064 (5)
C16	0.0234 (7)	0.0194 (6)	0.0125 (6)	0.0036 (5)	0.0050 (5)	0.0025 (5)
C17	0.0174 (6)	0.0138 (6)	0.0135 (6)	0.0009 (5)	0.0045 (5)	0.0014 (5)
C18	0.0140 (6)	0.0137 (6)	0.0155 (6)	0.0039 (5)	0.0030 (5)	0.0024 (5)
C19	0.0137 (6)	0.0146 (6)	0.0161 (6)	0.0037 (5)	0.0033 (5)	0.0024 (5)
C20	0.0149 (6)	0.0162 (6)	0.0178 (6)	−0.0001 (5)	0.0036 (5)	0.0013 (5)
O1W	0.0149 (5)	0.0254 (5)	0.0154 (5)	0.0058 (4)	0.0049 (4)	0.0004 (4)
C21	0.0190 (6)	0.0185 (6)	0.0188 (7)	0.0015 (5)	0.0071 (5)	0.0039 (5)
C22	0.0199 (6)	0.0178 (6)	0.0153 (6)	0.0055 (5)	0.0047 (5)	0.0003 (5)
C23	0.0242 (7)	0.0156 (6)	0.0194 (7)	−0.0032 (5)	0.0044 (5)	−0.0018 (5)
C24	0.0235 (7)	0.0159 (6)	0.0183 (7)	−0.0019 (5)	0.0068 (5)	0.0013 (5)
O2W	0.0120 (4)	0.0137 (4)	0.0085 (4)	0.0000 (3)	0.0036 (3)	0.0012 (3)

### Geometric parameters (Å, º)

**Table:**

Co1—O4	2.0494 (9)	C8—H8	0.9300
Co1—O1W	2.0743 (10)	C9—C10	1.385 (2)
Co1—N2	2.1038 (11)	C9—H9	0.9300
Co1—N3	2.1039 (11)	C10—C11	1.386 (2)
Co1—O2W	2.1394 (9)	C10—H10	0.9300
Co1—O2W^i^	2.1617 (9)	C11—C12	1.3908 (17)
O1—N1	1.2282 (16)	C11—H11	0.9300
N1—O2	1.2262 (15)	C12—C13	1.4868 (18)
N1—C1	1.4712 (16)	C13—C14	1.3960 (18)
C1—C2	1.3833 (18)	C14—C15	1.3845 (19)
C1—C6	1.3851 (19)	C14—H14	0.9300
N2—C8	1.3376 (17)	C15—C16	1.3861 (19)
N2—C12	1.3550 (16)	C15—H15	0.9300
C2—C3	1.3857 (18)	C16—C17	1.3851 (18)
C2—H2	0.9300	C16—H16	0.9300
O3—C7	1.2581 (16)	C17—H17	0.9300
N3—C17	1.3386 (16)	C18—C19	1.5176 (18)
N3—C13	1.3511 (16)	C19—C24	1.3906 (18)
C3—C4	1.3918 (18)	C19—C20	1.3934 (18)
C3—H3	0.9300	C20—C21	1.3890 (19)
O4—C7	1.2655 (15)	C20—H20	0.9300
N4—O7	1.2251 (16)	O1W—H1A	0.81 (2)
N4—O8	1.2284 (16)	O1W—H1B	0.82 (2)
N4—C22	1.4763 (17)	C21—C22	1.3831 (19)
C4—C5	1.3951 (17)	C21—H21	0.9300
C4—C7	1.5121 (17)	C22—C23	1.3844 (19)
C5—C6	1.3900 (18)	C23—C24	1.390 (2)
C5—H5	0.9300	C23—H23	0.9300
O5—C18	1.2590 (16)	C24—H24	0.9300
C6—H6	0.9300	O2W—H2A	0.79 (2)
O6—C18	1.2499 (16)	O2W—H2B	0.84 (2)
C8—C9	1.3897 (19)		
			
O4—Co1—O1W	88.78 (4)	C9—C10—C11	119.35 (12)
O4—Co1—N2	95.06 (4)	C9—C10—H10	120.3
O1W—Co1—N2	98.21 (4)	C11—C10—H10	120.3
O4—Co1—N3	172.65 (4)	C10—C11—C12	119.11 (12)
O1W—Co1—N3	89.91 (4)	C10—C11—H11	120.4
N2—Co1—N3	77.97 (4)	C12—C11—H11	120.4
O4—Co1—O2W	87.29 (4)	N2—C12—C11	121.70 (12)
O1W—Co1—O2W	93.78 (4)	N2—C12—C13	115.43 (11)
N2—Co1—O2W	167.83 (4)	C11—C12—C13	122.86 (12)
N3—Co1—O2W	100.02 (4)	N3—C13—C14	121.59 (12)
O4—Co1—O2W^i^	91.60 (4)	N3—C13—C12	115.34 (11)
O1W—Co1—O2W^i^	172.46 (4)	C14—C13—C12	123.07 (11)
N2—Co1—O2W^i^	89.26 (4)	C15—C14—C13	118.67 (12)
N3—Co1—O2W^i^	90.64 (4)	C15—C14—H14	120.7
O2W—Co1—O2W^i^	78.72 (4)	C13—C14—H14	120.7
O2—N1—O1	123.42 (12)	C14—C15—C16	119.70 (12)
O2—N1—C1	118.33 (11)	C14—C15—H15	120.2
O1—N1—C1	118.23 (11)	C16—C15—H15	120.2
C2—C1—C6	123.04 (12)	C17—C16—C15	118.36 (13)
C2—C1—N1	117.76 (12)	C17—C16—H16	120.8
C6—C1—N1	119.15 (11)	C15—C16—H16	120.8
C8—N2—C12	118.50 (11)	N3—C17—C16	122.72 (12)
C8—N2—Co1	126.05 (9)	N3—C17—H17	118.6
C12—N2—Co1	115.11 (8)	C16—C17—H17	118.6
C1—C2—C3	117.98 (12)	O6—C18—O5	125.68 (12)
C1—C2—H2	121.0	O6—C18—C19	119.05 (12)
C3—C2—H2	121.0	O5—C18—C19	115.27 (11)
C17—N3—C13	118.93 (11)	C24—C19—C20	119.42 (12)
C17—N3—Co1	125.64 (9)	C24—C19—C18	120.96 (12)
C13—N3—Co1	115.41 (8)	C20—C19—C18	119.60 (12)
C2—C3—C4	120.82 (12)	C21—C20—C19	120.64 (12)
C2—C3—H3	119.6	C21—C20—H20	119.7
C4—C3—H3	119.6	C19—C20—H20	119.7
C7—O4—Co1	129.34 (8)	Co1—O1W—H1A	116.9 (15)
O7—N4—O8	123.35 (12)	Co1—O1W—H1B	127.0 (15)
O7—N4—C22	118.32 (12)	H1A—O1W—H1B	114 (2)
O8—N4—C22	118.33 (12)	C22—C21—C20	118.29 (13)
C3—C4—C5	119.66 (12)	C22—C21—H21	120.9
C3—C4—C7	119.19 (11)	C20—C21—H21	120.9
C5—C4—C7	121.15 (11)	C21—C22—C23	122.70 (13)
C6—C5—C4	120.51 (12)	C21—C22—N4	117.93 (12)
C6—C5—H5	119.7	C23—C22—N4	119.34 (12)
C4—C5—H5	119.7	C22—C23—C24	117.98 (13)
C1—C6—C5	117.98 (12)	C22—C23—H23	121.0
C1—C6—H6	121.0	C24—C23—H23	121.0
C5—C6—H6	121.0	C23—C24—C19	120.94 (13)
O3—C7—O4	126.09 (11)	C23—C24—H24	119.5
O3—C7—C4	118.54 (11)	C19—C24—H24	119.5
O4—C7—C4	115.36 (11)	Co1—O2W—Co1^i^	101.28 (4)
N2—C8—C9	122.98 (12)	Co1—O2W—H2A	100.6 (16)
N2—C8—H8	118.5	Co1^i^—O2W—H2A	110.0 (16)
C9—C8—H8	118.5	Co1—O2W—H2B	121.6 (14)
C10—C9—C8	118.37 (13)	Co1^i^—O2W—H2B	112.2 (14)
C10—C9—H9	120.8	H2A—O2W—H2B	110 (2)
C8—C9—H9	120.8		

Symmetry code: (i) −*x*+1, −*y*+1, −*z*+1.

### Hydrogen-bond geometry (Å, º)

**Table:**

*D*—H···*A*	*D*—H	H···*A*	*D*···*A*	*D*—H···*A*
O2*W*—H2*B*···O5^ii^	0.84 (2)	1.67 (2)	2.5101 (13)	173 (2)
O2*W*—H2*A*···O3	0.79 (2)	1.88 (2)	2.6483 (13)	164 (2)
O1*W*—H1*B*···O3^iii^	0.82 (2)	2.04 (2)	2.8477 (14)	174 (2)
O1*W*—H1*A*···O6^i^	0.81 (2)	1.88 (2)	2.6803 (14)	171 (2)
C21—H21···O2^iv^	0.93	2.48	3.2219 (17)	137
C17—H17···O5^ii^	0.93	2.24	3.1679 (16)	172
C16—H16···O2^v^	0.93	2.57	3.4644 (17)	160
C14—H14···O6^vi^	0.93	2.41	3.3126 (16)	164
C9—H9···O7^vii^	0.93	2.64	3.5467 (18)	164

Symmetry codes: (i) −*x*+1, −*y*+1, −*z*+1; (ii) *x*−1, *y*, *z*; (iii) −*x*, −*y*+1, −*z*+1; (iv) *x*+1, *y*, *z*−1; (v) *x*, *y*, *z*−1; (vi) *x*, *y*+1, *z*; (vii) *x*, *y*+1, *z*+1.
